# Facial mask masking tardive dyskinesia

**DOI:** 10.1192/j.eurpsy.2021.833

**Published:** 2021-08-13

**Authors:** I. Ganhao

**Affiliations:** Clinic 6, Centro Hospitalar Psiquiatrico de Lisboa, Quinta do Anjo, Portugal

**Keywords:** facial masks, COVID-19, tardive dyskinesia

## Abstract

**Introduction:**

Facial covering and mask use is generally considered a preventive measure in reducing spread of infectious respiratory illnesses. With the COVID-19 pandemic, covering of the face, except the eyes, has become the norm for the first time for most people. Social interactions and clinical observation rely heavily on non-verbal communication of which facial expression is of utmost importance. While clinicians, especially in mental health settings, are acutely aware of the loss of information transmitted through the lower half of the face, signs of tardive dyskinesia may be forgotten in the list of potentially missed information.

**Objectives:**

To reflect on possible failure to detect orobuccolingual movements of tardive dyskinesia due to use of facial masks.

**Methods:**

Reflection on a clinical case of a patient with a treatment refractory psychosis who presents to an outpatient appointment with a facial mask. After the appointment, a family member transmitted having observed what appeared to be involuntary masticatory movements in the patient.

**Results:**

Facial masks and coverings occult signs that may be visible on the lower half of the face.

**Conclusions:**

Facial masks and coverings are essential in preventing COVID-19 contagion. Clinicians must keep in mind loss of information when part of the face is not visible. Tardive dyskinesia with orobuccolingual movements may be missed behind a mask. Family or other people who cohabit with the patient are essential information providers.

